# A Novel Circular RNA Generated by FGFR2 Gene Promotes Myoblast Proliferation and Differentiation by Sponging miR-133a-5p and miR-29b-1-5p

**DOI:** 10.3390/cells7110199

**Published:** 2018-11-06

**Authors:** Xiaolan Chen, Hongjia Ouyang, Zhijun Wang, Biao Chen, Qinghua Nie

**Affiliations:** 1 Department of Animal Genetics, Breeding and Reproduction, College of Animal Science, South China Agricultural University, Guangzhou 510642, China; xiaolanchen@stu.scau.edu.cn (X.C.); oyolive@stu.scau.edu.cn (H.O.); zhijunwang@stu.scau.edu.cn (Z.W.); biaochen@stu.scau.edu.cn (B.C.); 2 National-Local Joint Engineering Research Center for Livestock Breeding, Guangdong Provincial Key Lab of Agro-Animal Genomics and Molecular Breeding, and the Key Lab of Chicken Genetics, Breeding and Reproduction, Ministry of Agriculture, Guangzhou 510642, China; 3 College of Animal Science & Technology, Zhongkai University of Agriculture and Engineering, Guangzhou 510225, China

**Keywords:** circular RNA, circFGFR2, *FGFR2*, miR-133a-5p, miR-29b-1-5p, skeletal muscle, proliferation, differentiation

## Abstract

It is well known that fibroblast growth factor receptor 2 (*FGFR2*) interacts with its ligand of fibroblast growth factor (*FGF*) therefore exerting biological functions on cell proliferation and differentiation. In this study, we first reported that the *FGFR2* gene could generate a circular RNA of circFGFR2, which regulates skeletal muscle development by sponging miRNA. In our previous study of circular RNA sequencing, we found that circFGFR2, generated by exon 3–6 of *FGFR2* gene, differentially expressed during chicken embryo skeletal muscle development. The purpose of this study was to reveal the real mechanism of how circFGFR2 affects skeletal muscle development in chicken. In this study, cell proliferation was analyzed by both flow cytometry analysis of the cell cycle and 5-ethynyl-2′-deoxyuridine (EdU) assays. Cell differentiation was determined by analysis of the expression of the differentiation marker gene and Myosin heavy chain (MyHC) immunofluorescence. The results of flow cytometry analysis of the cell cycle and EdU assays showed that, overexpression of circFGFR2 accelerated the proliferation of myoblast and QM-7 cells, whereas knockdown of circFGFR2 with siRNA reduced the proliferation of both cells. Meanwhile, overexpression of circFGFR2 accelerated the expression of myogenic differentiation 1 (*MYOD*), myogenin (*MYOG*) and the formation of myotubes, and knockdown of circFGFR2 showed contrary effects in myoblasts. Results of luciferase reporter assay and biotin-coupled miRNA pull down assay further showed that circFGFR2 could directly target two binding sites of miR-133a-5p and one binding site of miR-29b-1-5p, and further inhibited the expression and activity of these two miRNAs. In addition, we demonstrated that both miR-133a-5p and miR-29b-1-5p inhibited myoblast proliferation and differentiation, while circFGFR2 could eliminate the inhibition effects of the two miRNAs as indicated by rescue experiments. Altogether, our data revealed that a novel circular RNA of circFGFR2 could promote skeletal muscle proliferation and differentiation by sponging miR-133a-5p and miR-29b-1-5p.

## 1. Introduction

Circular RNA is a large class of endogenous RNA with a covalently closed loop. It was actually discovered in plants, mouse, and yeast twenty years ago [1,2,3]. However, it has been regarded as unvalued mis-splicing product of mRNA in the last decades as a few kinds and a small quantity of circular RNAs have been found [4]. In addition, circular RNA has no 5′ caps and 3′ tails, and it could be easily abandoned by traditional sequencing technology [5]. Fortunately, with the rapid development of high throughput sequencing technology, the mysterious veil of circular RNA was revealed step by step [6]. Large amounts of circular RNAs were discovered in many species, including human [7], monkey [8], and pig [9].

Nowadays, circular RNA is considered as an up-rising star in the small RNAs interaction network with regulatory potency [10]. The diverse functions of circular RNA act as miRNA sponge, participating in regulating the expression of its own linear RNA in different ways [10,11], sequestering proteins [12,13], coding protein in vitro [14,15,16], and deriving pseudogenes [17]. Acting as miRNA sponge is a well-studied function of circular RNA, also known as a competing endogenous RNA mechanism (ceRNA). The CeRNA mechanism is that messenger RNAs, transcribed pseudogenes, and long noncoding RNAs competitively combine with the same miRNA response elements (MREs), and then eliminate the inhibition of miRNA on their target genes. Circular RNA interacted with miRNA are ubiquitous in a variety of tissues. A well-known example is that ciRS-7 has more than 70 highly conserved target sites of miR-7 and can extremely repress the activity of miR-7 [18]. This is the strongest evidence for a circRNA function as the miRNA sponge has thrust circRNAs into the spotlight and spurred a multitude of studies searching for functional circRNA sponges [19,20,21].

In previous work [22], we used leg muscle tissues of two female XingHua chickens from each at days E11, E16, and P1 for circRNA sequencing to comprehensively identify stably expressed circRNAs during skeletal muscle development at the embryonic stage. As a result, 13,377 potential circRNAs were identified and abundantly expressed among different development stages. Furthermore, the differentially expressed genes (DEGs) analysis showed 462 of them were differentially expressed at different development stages. CircFGFR2 was one of the DEcircRNAs with high expression during skeletal muscle development. Through divergent reverse-transcription PCR and RNase R treatment, in previous work [22], we confirmed that circFGFR2 was a stable exonic circular RNA formed by 3–6 exons of fibroblast growth factor receptor 2 (*FGFR2*), with a length of 636 bp. As a member of *FGFRs* family, *FGFR2* interacts with fibroblast growth factor (*FGF*) to exert biological effects on cell proliferation and differentiation as well as skeletal development [23]. The different expression level of circRNAs implied that they could potentially regulate skeletal muscle development. We previously revealed that circRBFOX2 could interact with miR-206 to regulate skeletal muscle cell proliferation and differentiation [22]. Considering all of that, we assumed that circFGFR2 was another candidate circRNA that probably affects skeletal muscle development.

In comparison to circular RNA, miRNAs are extremely well studied non-coding RNAs that suppress protein expression by targeting the 3′-UTR (Untranslated Region) of their mRNA with Argonaute effector protein [24,25]. The MiR-133 family has two members of miR-133a and miR-133b, which are found to specifically express in skeletal muscle and cardiac [26]. MiR-133a-5p belongs to the miR-133a cluster. Many studies have shown that miR-133 families are involved in regulating the proliferation and differentiation of various kinds of skeletal muscle cells [27,28]. However, the role of miR-133a-5p on skeletal muscle development has not been reported in poultry. MiR-29b-1-5p is a mature miRNA and belongs to the miR-29b cluster of the miR-29 family. This family has other clusters of miR-29a and miR-29c [29]. In chicken, the gga-miR-29b cluster contains gga-miR-29b-1-5p, gga-miR-29b-2-5p, and gga-miR-1701. MiR-29s are efficient regulators in the process of cell proliferation [30], differentiation [31], apoptosis [32,33,34] as well as DNA methylation [35,36] in different cell types. In skeletal muscle, miR-29s could participate in regulating skeletal myogenesis through different pathways. In mouse C2C12 cells, they could down-regulate *Rybp* (Ring1 and YY1-binding Protein) [37], AKT serine/threonine kinase 3 (*AKT3*) [38], and histone deacetylase 4 (*HDAC4*) [39] to regulate the differentiation of skeletal muscle cell. In addition, miR-29s were also related to some muscle diseases, including muscle atrophy [40], dystrophic muscle pathogenesis [41], and Duchenne muscular dystrophy [42]. Obviously, miR-29s play important roles in muscle development.

In this study, we aim to investigate the effects of circFGFR2 on skeletal muscle cell development, and to reveal its regulatory mechanism by interacting miR-133a-5p and miR-29b-1-5p.

## 2. Materials and Methods

### 2.1. Ethics Statement

This study was carried out in accordance with the principles of the Basel Declaration and recommendations of the Statute on the Administration of Laboratory Animals, the South China Agriculture University Institutional Animal Care and Use Committee. The protocol was approved by the South China Agriculture University Institutional Animal Care and Use Committee (approval, 19 November 2017, ID: 2017046).

### 2.2. Primers

All primers used in this study were designed by Premier Primer 5.0 software (Premier Bio-soft International, Palo Alto, CA, USA) and synthesized by Sangon (Sangon Biotech, Shanghai, China). The detailed information of all primers is listed in Table 1.

### 2.3. RNA Extraction, cDNA Synthesis and Quantitative Real-Time PCR

The total RNA was extracted from cells by using RNAiso reagent (TaKaRa, Otsu, Japan). The quality and concentration of all obtained RNA samples were determined by 1.5% agarose gel electrophoresis and evaluated for optical density 260/280 ratio by Nanodrop 2000 spectrophotometer (Thermo, Waltham, MA, USA). For mRNA and circFGFR2 expression analysis, cDNA synthesis for mRNA was performed using a PrimeScript RT Reagent Kit (Perfect Real Time) (TaKaRa, Otsu, Japan). The *β-actin* gene was used as an internal control for quantitative real-time PCR (qRT-PCR) analysis. The reverse transcription reaction for miRNA was performed using ReverTra Ace qPCR RT Kit (Toyobo, Osaka, Japan). The specific Bulge-loop miRNA qRT-PCR Primer for miR-133a-5p, miR-29b-1-5p and U6 were designed by RiboBio (RiboBio, Guangzhou, China). qRT-PCR was performed on a Bio-Rad CFX96 Real-Time Detection System (Bio-Rad, Hercules, CA, USA) using iTaq™ Universal SYBR^®^ Green Supermix Kit (Bio-Rad, Hercules, CA, USA). Each sample was assayed in triplicate, following the manufacturer’s instructions. The specificity of the product was evaluated by the melting curve, and the quantitative values were obtained from the threshold PCR cycle number (Ct) at which the increase in signal is associated with an exponential growth at which the PCR product starts to be detected. The relative mRNA level in each sample was indicated by 2*^−^*^ΔΔ*C*t^.

### 2.4. RNA Oligonucleotides and Plasmids Construction

The gga-miR-133a-5p mimic, gga-miR-29b-1-5p mimic and mimic control duplexes, the 3′ end biotinylated gga-miR-133a-5p, gga-miR-29b-1-5p and mimic control duplexes, siRNA target against circFGFR2 (si-circFGFR2, 5′-CGATGTTGTCGAGCCGCCA-3′) and non-specific siRNA negative control were synthesized by RiboBio (Guangzhou, China). For circFGFR2 overexpression plasmids construction, the linear sequences of circFGFR2 was amplified by PCR with primer *FGFR2-2*, and the cDNA template was synthesized from the RNA of chicken primary myoblast by RT-PCR. Then, the obtained linear sequences were cloned into *KpnI* and *BamHI* restriction sites of a circular expression vector-the pCD2.1-ciR vector (Geneseed Biotech, Guangzhou, China) according to the manufacturer’s protocol, so as to generate the pCD2.1-circFGFR2 overexpression vector. For pmirGLO dual-luciferase reporter construction: the whole linear sequences of circFGFR2 were cloned into *XhoI and SalI* restriction sites of pmirGLO vector to generate the wild reporter vector (PGLO-WT reporter vector), which includes the predicted binding sites of miR-133a-5p and miR-29b-1-5p. PGLO-MT1 and PGLO-MT2 were two mutational reporter vectors of miR-133a-5p which were cloned into *XhoI and SalI* restriction sites of pmirGLO vector by PCR mutagenesis. We changed one of miR-133a-5p binding seed sequences from “CCAG” to “TTGA” in PGLO-MT1, while in PGLO-MT2 we changed another miR-133a-5p binding seed sequence (which included the binding site of miR-29b-1-5p) from “CCAG” to “GTTG”. All luciferase reporters were constructed by Hongxun Biotech (Suzhou, China).

### 2.5. Cell Culture

Chicken embryo fibroblast cell line (DF-1) cells were cultured in high-glucose Dulbecco’s modified Eagle’s medium (Gibico, Grand Island, NY, USA) with 10% (*v*/*v*) fetal bovine serum (FBS) (Gibco, Grand Island, NY, USA) and 0.2% penicillin/streptomycin (Invitrogen, Carlsbad, CA, USA). Quail muscle cell line (QM-7) cells were cultured in high-glucose M199 medium (Gibco, USA) with 10% (*v*/*v*) FBS, 10% tryptose phosphate broth solution (Sigma, Louis, MO, USA) and 0.2% penicillin/streptomycin (Invitrogen, Carlsbad, CA, USA). Chicken primary myoblasts were isolated from the leg muscles of 11-day embryo age (E11) chickens. Leg tissues were collected from E11 chickens by completely removing skin and bones. Leg muscle was minced into sections of approximately 1 mm with scissors and then digested with 0.25% trypsin (Gibco, Grand Island, NY, USA) at 37 °C in a shaking water bath (90 oscillations/min) for 20 min. Digestions were terminated by adding equal values of complete medium-(RPMI)1640 medium with 20% FBS, 1% nonessential amino acids and 0.2% penicillin/streptomycin (Invitrogen, Carlsbad, CA, USA). The mixture was filtered through a nylon mesh with 70 mm pores (BD Falcon, Greiner, Germany). The filtered cells were centrifuged at 500× *g* for 5 min, and maintained in complete medium at 37 °C in a 5% CO_2_, humidified atmosphere. Serial plating was performed to enrich myoblasts and to remove fibroblasts.

### 2.6. Transfections

Transfections were performed with Lipofectamine 3000 reagent (Invitrogen, Carlsbad, CA, USA) according to the manufacturer’s instruction. Nucleic acids were diluted in OPTI-MEM Medium (Gibco, Grand Island, NY, USA).

### 2.7. 5-Ethynyl-2′-Deoxyuridine (EdU) Assays

After cells were transfected for 48 h, myoblasts were exposed to 50 μM 5-ethynyl-2′-deoxyuridine (EdU) (RiboBio, Guangzhou, China) for 2 h at 37 °C. Next, the cells were fixed in 4% paraformaldehyde (PFA) for 30 min and 2 mg/mL glycine solution was used to neutralize the 4% PFA. Cells were, then, permeabilized with 0.5% Triton X-100. Subsequently, 1× Apollo reaction cocktail (RiboBio, Guangzhou, China) was added to the cells and incubated for 30 min. The cells were stained with Hoechst 33342 for 30 min for DNA content analysis. Finally, the EdU-stained cells were visualized under a fluorescence microscope (Nikon, Tokyo, Japan or Leica, Wetzlar, Germany). The analysis of myoblast proliferation (ratio of EdU+ to all myoblasts) was performed using images of randomly selected fields obtained on the fluorescence microscope.

### 2.8. Flow Cytometry Analysis of the Cell Cycle

Myoblast cultures in growth medium (GM) were collected after a 48 h or 36 h-transfection and then fixed in 70% ethanol overnight at −20 °C. After incubation in 50 μg/mL propidium iodide (PI) (Sigma, Louis, MO, USA) containing 10 μg/mL RNase A (TaKaRa, Otsu, Japan) and 0.2% (*v*/*v*) Triton X-100 (Sigma, Louis, MO, USA) for 30 min at 4 °C, the cells were analyzed by using a BD AccuriC6 flow cytometer (BD Biosciences, San Jose, CA, USA) and FlowJo7.6 software (Treestar Incorporated, Ashland, OR, USA).

### 2.9. Immunofluorescence

For immunofluorescence, cells were seeded in 24-well plates. After transfection for 48 h, cells were fixed in 4% formaldehyde for 20 min then washed three times with PBS for 5 min. Subsequently, the cells were permeabilized by adding 0.1% Triton X-100 for 5 min and blocked with goat serum for 30 min. After incubation with MyHC (B103; DSHB, Iowa City, IA, USA; 0.5 μg/mL) at 37 °C for 2 h, the Fluorescein (FITC)-conjugated AffiniPure Goat Anti-Mouse IgG (H + L) (Bioworld, Minneapolis, MN, USA; 1:200) or FITC (Bioworld, Minneapolis, MN, USA; 1:50) was added and the cells were incubated at room temperature for 1 h. The cell nuclei were stained with 4′,6-diamidino-2-phenylindole (DAPI, Beyotime, Shanghai, China; 1:50) for 5 min. Images were obtained with a fluorescence microscope (Leica, Wetzlar, Germany). The area of cells labeled with anti-MyHC was measured by using ImageJ software (National Institutes of Health, Bethesda, MD, USA), and the total myotube area was calculated as a percentage of the total image area covered by myotubes.

### 2.10. Luciferase Reporter Assay

To investigate the binding sites of circFGFR2 with miR-133a-5p/miR-29b-1-5p, DF-1 cells were seeded in 96-well plates and then co-transfected with 100 ng of PGLO-WT reporter vector or mutant vectors PGLO-MT1 or PGLO-MT2, and 50 nM of miR-133a-5p/miR-29b-1-5p mimics or mimic control duplexes by using Lipofectamine 3000 reagent (Invitrogen, Carlsbad, CA, USA). To investigate whether circFGFR2 could inhibit the activity of miR-133a-5p/miR-29b-1-5p, DF-1 cells were seeded in 96-well plates and then co-transfected with 100 ng of PGLO-WT reporter vector or circFGFR2 overexpression vector, and 50 nM of miR-133a-5p/miR-29b-1-5p mimics or mimic control duplexes by using Lipofectamine 3000 reagent (Invitrogen, Carlsbad, CA, USA). After 48 h post transfection, luciferase activity analysis was performed using a Fluorescence/Multi-Detection Microplate Reader (BioTek, Winooski, VT, USA) and a Dual-GLO^®^ Luciferase Assay System Kit (Promega, Madison, WI, USA). Firefly luciferase activities were normalized to Renilla luminescence in each well.

### 2.11. Biotin-Coupled miRNA Pull Down Assay

Transfection procedure: the 100 nM of 3′ end biotinylated miR-133a-5p, miR-29b-1-5p or mimic NC (RiboBio, Guangzhou, China) were transfected into QM-7 cells along with 30 µg circFGFR2 expression vector in T75 cell culture bottle. At 24 h after transfection, the cells were harvested and washed in PBS, then lysed in lysis buffer. A total of 100 μL washed streptavidin magnetic beads were blocked for 2 h and then added to each reaction tube to pull down the biotin-coupled RNA complex. All the tubes were incubated for 4 h on a rotator at a low speed (10 r/min). The beads were washed with lysis buffer five times and RNAiso reagent (TaKaRa, Otsu, Japan) was used to recover RNAs specifically interacting with miRNA. The abundance of circFGFR2 in bound fractions was evaluated by qRT-PCR analysis.

### 2.12. Statistical Analysis

In all panels, results are expressed as the mean ± S.E.M. of three independent experiments. For two group comparison analysis, statistical significance of differences between means was analyzed by unpaired Student’s *t*-test. For multiple comparison analysis, data were analyzed by one-way ANOVA followed by both least significant difference (LSD) and Duncan test through Statistical Package for the Social Sciences software (SPSS 17.0, Chicago, IL, USA). We considered *p* < 0.05 to be statistically significant. * p < 0.05; ** p < 0.01. NC, negative control.

## 3. Results

### 3.1. CircFGFR2 Promotes Myoblast Proliferation

**Figure 1:**
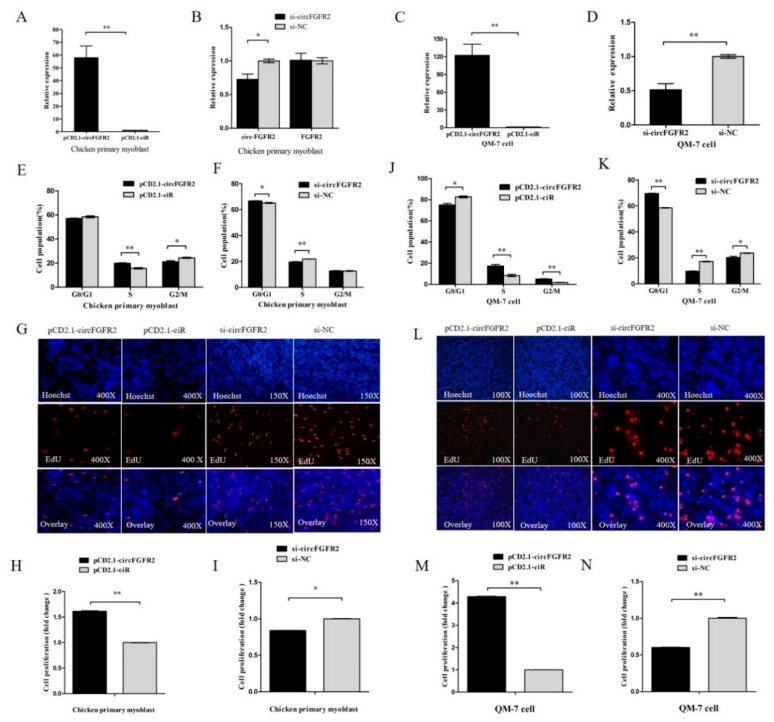
CircFGFR2 promotes myoblast proliferation. (**A**,**B**) The relative expression of circFGFR2 after transfected chicken primary myoblasts with 1 µg pCD2.1-circFGFR2 or 50 nM si-circFGFR2 for 48 h. (**C**,**D**) The relative expression of circFGFR2 after transfected QM-7 cells with 1 µg pCD2.1-circFGFR2 or 50nM si-circFGFR2 for 48 h. (**E**,**F**) Cell cycle analysis of chicken primary myoblasts transfected with 1 µg circFGFR2 pCD2.1-circFGFR2 or 50 nM si-circFGFR2 for 36 h. (**G**) 5-ethynyl-2′-deoxyuridine (EdU) assays for chicken primary myoblasts transfected with 1 µg circFGFR2 pCD2.1-circFGFR2 or 50 nM si-circFGFR2 for 36 h. (**H**,**I**) The percentage of EdU-stained chicken primary myoblasts after overexpression or knockdown of circFGFR2 for 36 h. (**J**,**K**) Cell cycle analysis of QM-7 cells transfected with 1 µg circFGFR2 pCD2.1-circFGFR2 or 50 nM si-circFGFR2 for 48 h. (**L**) EdU assays for QM-7 cells transfected with 1 µg circFGFR2 pCD2.1-circFGFR2 or 50 nM si-circFGFR2 for 48 h. (**M**,**N**) The percentage of EdU-stained chicken primary myoblasts after overexpression or knockdown of circFGFR2 for 48 h. In all panels, the results are shown as mean ± S.E.M., and the data are represented by three independent assays. Statistical significance of differences between means was assessed using an unpaired Student’s *t*-test (* *p* < 0.05; ** *p* < 0.01).

**Figure 2:**
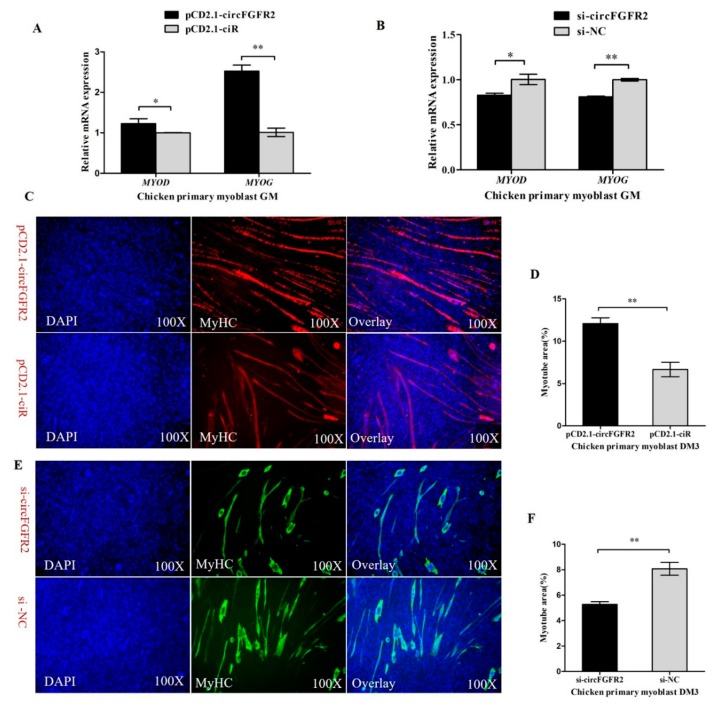
CircFGFR2 promotes myoblast differentiation. (**A**) Overexpression of circFGFR2 promotes mRNA expression of MYOD and MYOG. (**B**) Knockdown of circFGFR2 inhibits the mRNA expression of MYOD and MYOG. (**C**,**D**) Overexpression of circFGFR2 facilitates the formation of myotubes. (**E**,**F**) Down-regulation of circFGFR2 suppresses the formation of myotubes. In all panels, data are presented as mean ± S.E.M. of three biological replicates. Statistical significance of differences between means was assessed using an unpaired Student’s *t*-test (* *p* < 0.05; ** *p* < 0.01).

To investigate the role of circFGFR2 in skeletal muscle cell proliferation, we conducted overexpression and knocked down experiments by transfecting circFGFR2 overexpression vector and siRNAs (pCD2.1-circFGFR2 and si-circFGFR2) into chicken primary myoblast and QM-7 cell. The relative expression of circFGFR2 was detected after 48 h post transfection by qRT-PCR. Result showed that both the effect of overexpression and knockdown had reached a significant level in both myoblast and QM-7 cell (Figure 1A–D), and si-circFGFR2 specifically downregulated the expression of circFGFR2 but not linear mRNA of *FGFR2* (Figure 2B). Furthermore, we detected the proliferation process of both chicken primary myoblast and QM-7 cell by flow cytometry for cell cycle analysis and 5-ethynyl-2′-deoxyuridine (EdU) incorporation assays after transfecting with pCD2.1-circFGFR2/pCD2.1-ciR, or si-circFGFR2/control. Cell cycle analysis showed that overexpression of circFGFR2 increased the cell population in S phase and decreased the cell population in G1/0 and G2/M phases (Figure 1E) while knockdown of circFGFR2 decreased the cell population in S phase and increased the cell population in G1/0 phase, as observed in chicken primary myoblast (Figure 1F). Meanwhile, the result of EdU strain assay showed that there were significantly more cells in the pCD2.1-circFGFR2 transfected group than in the control group (Figure 1G,H), whereas knockdown of circFGFR2 significantly decreased the numbers of EdU strained cells (Figure 1G,I). These results indicated that circFGFR2 could promote the proliferation rate of chicken primary myoblast. As expected, we obtained similar results in QM-7 cell (Figure 1J–N). These results suggested that circFGFR2 could significantly promote the proliferation of myoblast and QM-7 cell.

### 3.2. CircFGFR2 Promotes Myoblast Differentiation

Myogenesis is a complex process including myoblast proliferation, differentiation and myotube formation and is controlled by myogenic regulatory factors (MRFs), *MYOD*, *MYOG*, myogenic factor 5 (*Myf5*), and myogenic factor 6 (*Myf6*, also known as myogenic regulatory factor 4, *MRF4*). These factors activate muscle-specific genes to coordinate myoblasts to terminally withdraw from the cell cycle and subsequently fuse into multinucleated myotubes [43]. Following proliferation, the initiation of terminal differentiation and fusion begins with the expression of myogenin, which together with *MYOD*, activates the muscle specific structural and contractile genes to stimulate myoblast differentiation [44]. To address the potential role of circFGFR2 in primary myoblast differentiation, the expression of differentiation marker genes, including *MYOG* and *MYOD* were analyzed by qRT-PCR after transfecting with pCD2.1-circFGFR2/pCD2.1-ciR, or si-circFGFR2/control. Result showed that overexpression of circFGFR2 significantly promoted the expression of *MYOD* and *MYOG* while knockdown of circFGFR2 significantly inhibited the expression of *MYOD* and *MYOG* (Figure 2A,B). It indicated that circFGFR2 may promote chicken primary myoblast differentiation. Subsequently, we induced chicken primary myoblast differentiation in vitro, as soon as the muscle cells started to differentiate into myotubes (the first day of differentiation, DM1), we transfected them with pCD2.1-circFGFR2/pCD2.1-ciR. MyHC immunofluorescence staining was carried out on the differentiated myoblasts after 36 h post transfection (DM3). According to the immunofluorescence staining, we found that the areas of myotubes of pCD2.1-circFGFR2 transfected group were prominently greater than that of the control group (Figure 2C,D). Conversely, the areas of myotubes in the si-circFGFR2 transfected group were lower than that of the control group (Figure 2E,F). The result showed that circFGFR2 could promote the formation of myotubes and promote the early differentiation of chicken primary myoblast.

### 3.3. CircFGFR2 Interacts with miR-133a-5p and miR-29b-1-5p, and Inhibits the Expression of miR-133a-5p and miR-29b-1-5p in Myoblast

**Figure 3:**
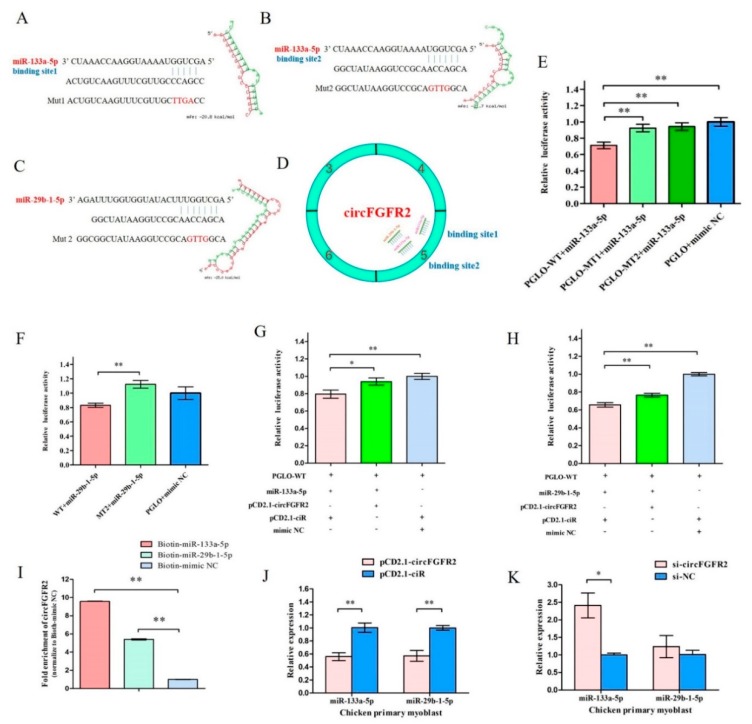
CircFGFR2 sponges with miR-133a-5p and miR-29b-1-5p, and inhibits the expression of miR-133a-5p and miR-29b-1-5p in myoblast. (**A**–**C**) The potential binding sites of miR-133a-5p and miR-29b-1-5p in circFGFR2. The mutant sequences in binding sites are highlighted in red. (**D**) A schematic drawing showing the putative binding sites of miR-133a-5p/miR-29b-1-5p associated with circFGFR2. (**E**,**F**) Luciferase assay was conducted by co-transfecting wild type or mutant linear sequence of circFGFR2 with miR-133a-5p/miR-29b-1-5p mimic or mimic-NC in DF-1 cells. (**G**,**H**) Luciferase assay was conducted by co-transfecting wild type circFGFR2 linear sequence and miR-133a-5p/miR-29b-1-5p mimic or mimic-NC and with circFGFR2 overexpression vector (pCD2.1-circFGFR2) or empty vector (pCD2.1-ciR). (**I**) Biotin-coupled miRNA pull down assay from the myoblast lysates after transfection with 3′ end biotinylated miR-133a-5p, miR-29b-1-5p or mimic NC. The expression level of circFGFR2 was quantified by qRT–PCR, and fold enrichment in the streptavidin captured fractions are plotted. (**J**,**K**) qRT–PCR analysis of the relative expression of miR-133a-5p and miR-29b-1-5p after overexpression or inhibition of circFGFR2. In all panels, results are expressed as the mean ± S.E.M. of three independent experiments. For two group comparison analysis, statistical significance of differences between means was analyzed by unpaired Student’s *t*-test. For multiple comparison analysis, data were analyzed by one-way ANOVA followed by both least significant difference (LSD) and Duncan test through SPSS software. We considered *p* < 0.05 to be statistically significant. * *p* < 0.05; ** *p* < 0.01. NC, negative control.

Circular RNA has been shown to act as miRNA sponge and circFGFR2 could promote myoblast proliferation and differentiation. We hypothesized that circFGFR2 exerts function by acting as miRNA sponge as well as regulating the expression of miRNA. To screen potential miRNAs that bind to circFGFR2, we used RNAhybrid to conduct the putative combination site between circFGFR2 and miR-133a-5p/miR-29b-1-5p. Interestingly, we found that circFGFR2 has two potential miR-133a-5p binding sites (binding site 1and binding site 2) and one potential miR-29b-1-5p binding site. The potential miR-29b-1-5p binding site shares six of seven nucleotides with the binding site 2 of miR-133a-5p. The mature sequence of miR-133a-5p/miR-29b-1-5p and the predicted binding sites of these two miRNAs are shown in Figure 3A–D.

To investigate the binding site of circFGFR2 with miR-133a-5p/miR-29b-1-5p, we constructed a dual-luciferase reporter by inserting the wild type (WT) or mutant (MT) linear sequence of circFGFR2 into the 3′ end of *firefly* luciferase of pmirGLO (PGLO) luciferase vector to generate a wild type reporter (PGLO-WT) and two mutant reporters (PGLO-MT1 and PGLO-MT2). PGLO-MT1 vector contains the mutated seed sequences for the binding site 1 of mir-133a-5p, and PGLO-MT2 contains the mutated seed sequence for miR-133a-5p binding site 2 and miR-29b-1-5p binding site. The mutant sequences are shown in Figure 3A–C. Then DF-1 cells were co-transfected with PGLO-WT, PGLO-MT1/PGLO-MT2/PGLO luciferase vector and co-transfected with miR-133a-5p/miR-29b-1-5p mimic/control duplexes, respectively. The relative luciferase activity in DF-1 cell line was significantly decreased when miR-133a-5p/miR-29b-1-5p mimic were co-transfected with PGLO-WT reporter (Figure 3E,F) compared with the miR-133a-5p/miR-29b-1-5p mimic and their correspondent mutant reporter co-transfected group. This result demonstrated that miR-133a-5p and miR-29b-1-5p could really combine with the predicted binding sites and miR-133a-5p could combine with both binding site 1 and site 2.

To study the effect of circFGFR2 on the activity of miR-133a-5p/miR-29b-1-5p, we conducted another luciferase reporter assay by co-transfected pCD2.1-circFGFR2 (circFGFR2 overexpression vector)/pCD2.1-ciR (the empty overexpression vector), miR-133a-5p/miR-29b-1-5p/mimic NC with PGLO-WT reporter vector. Luciferase reporter assay showed that the relative luciferase activity was significantly decreased when cells were co-transfected miR-133a-5p/miR-29b-1-5p mimic with PGLO-WT reporter, while the relative luciferase activity was significantly increased when cells were co-transfected the miR-133a-5p/miR-29b-1-5p mimic with pCD2.1-circFGFR2 (Figure 3G,H). It suggested that circFGFR2 could combine with exogenetic miR-133a-5p and miR-29b-1-5p and eliminate the activity of both miRNAs.

Subsequently, we also conducted biotin-coupled miRNA pull down assay to further confirm the interaction between circFGFR2 and miR-133a-5p/miR-29b-1-5p by using biotin-coupled miR-133a-5p and miR-29b-1-5p mimics. Compared with the negative control, we observed more than 8-fold enrichment of circFGFR2 in miR-133a-5p-captured fraction and more than 5-fold enrichment of circFGFR2 in miR-29b-1-5p-captured fraction (Figure 3I), which demonstrated that circFGFR2 could directly sponge miR-133a-5p and miR-29b-1-5p. The greater enrichment observed in miR-133a-5p-captured fraction is probably due to the fact that circFGFR2 contained two binding sites for miR-133a-5p but only one for miR-29b-1-5p.

In addition, the qRT-PCR result showed that overexpression of circFGFR2 could significantly decrease the expression of miR-133a-5p and miR-29b-1-5p (Figure 3J), while knockdown of circFGFR2 could up-regulate the expression of miR-133a-5p and miR-29b-1-5p in chicken primary myoblast (Figure 3K).

### 3.4. MiR-133a-5p and miR-29b-1-5p Inhibit Myoblast Proliferation

**Figure 4:**
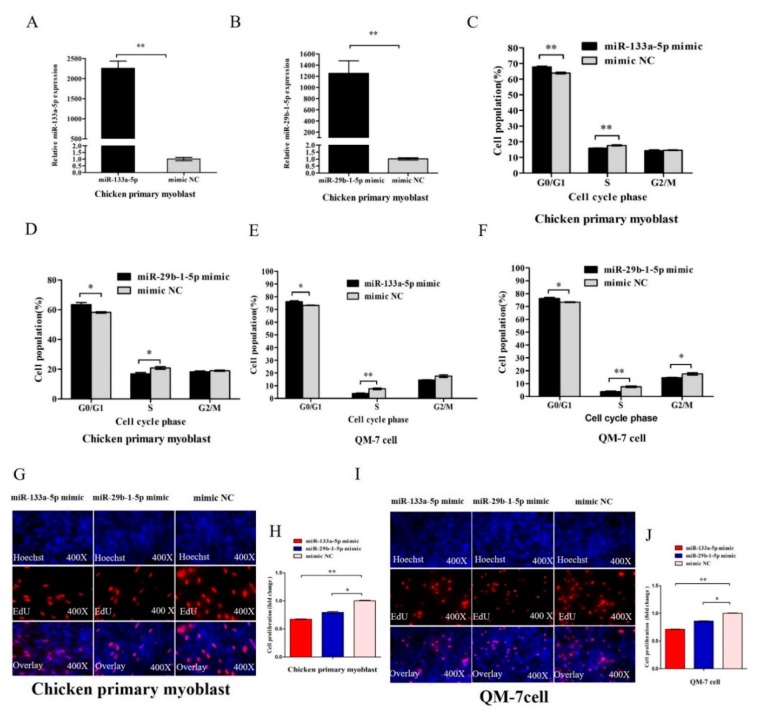
miR-133a-5p and miR-29b-1-5p inhibit myoblast proliferation. (**A**,**B**) The relative expression of miR-133a-5p and miR-29b-1-5p after transfected chicken primary myoblast with 50 nM miR-133a-5p and miR-29b-1-5p mimic for 48 h. (**C**,**D**) Cell cycle analysis of chicken primary myoblasts transfected with 50 nM miR-133a-5p and miR-29b-1-5p mimic for 36 h. (**E**,**F**) Cell cycle analysis of QM-7 cell transfected with 50 nM miR-133a-5p and miR-29b-1-5p mimic for 48 h. (**G**,**H**) EdU assay of chicken primary myoblasts transfected with 50 nM miR-133a-5p or miR-29b-1-5p mimic for 36 h. (**I**,**J**) EdU assay of QM-7 cell transfected with 50 nM miR-133a-5p or miR-29b-1-5p mimic for 48 h. In all panels, results are expressed as the mean ± S.E.M. of three independent experiments, and statistical significance of differences between means was assessed using an unpaired Student’s *t*-test (* *p* < 0.05; ** *p* < 0.01). NC, negative control.

As circFGFR2 had an effect on myoblast proliferation, we also confirmed that circFGFR2 could inhibit the expression and activity of miR-133a-5p and miR-29b-1-5p. We speculated that miR-133a-5p and miR-29b-1-5p had a potential effect on myoblast proliferation. To confirm our hypothesis, we synthesized miR-133a-5p and miR-29b-1-5p mimic. In chicken primary myoblast, we detected the expression of miR-133a-5p and miR-29b-1-5p after transfected chicken primary myoblast with 50 nM miR-133a-5p or miR-29b-1-5p mimic for 48 h. The expression of miR-133a-5p or miR-29b-1-5p was significantly increased by mimic (Figure 4A,B). Subsequently, in chicken primary myoblast, flow cytometry analysis showed that overexpression of miR-133a-5p or miR-29b-1-5p could prominently increase the numbers of cells that progressed to G0/G1 and reduced the numbers of S phase cells (Figure 4C,D). Meanwhile, we found similar results in QM-7 cells as indicated by cycle analysis. MiR-133a-5p or miR-29b-1-5p overexpression significantly increased the number of QM-7 cells that progressed to G0/G1 and reduced the number of S phase cells (Figure 4E,F). Furthermore, the EdU assay demonstrated that overexpression of miR-133a-5p and miR-29b-1-5p dramatically decreased the numbers of EdU strained cells (Figure 4G–J) in both chicken primary myoblast and QM-7 cell, which indicated that miR-133a-5p and miR-29b-1-5p could inhibit the proliferation rate of skeletal muscle cells. These results revealed that miR-133a-5p and miR-29b-1-5p could suppress myoblast proliferation.

### 3.5. CircFGFR2 Eliminates the Inhibition Effect of miR-133a-5p and miR-29b-1-5p on Myoblast Proliferation

**Figure 5:**
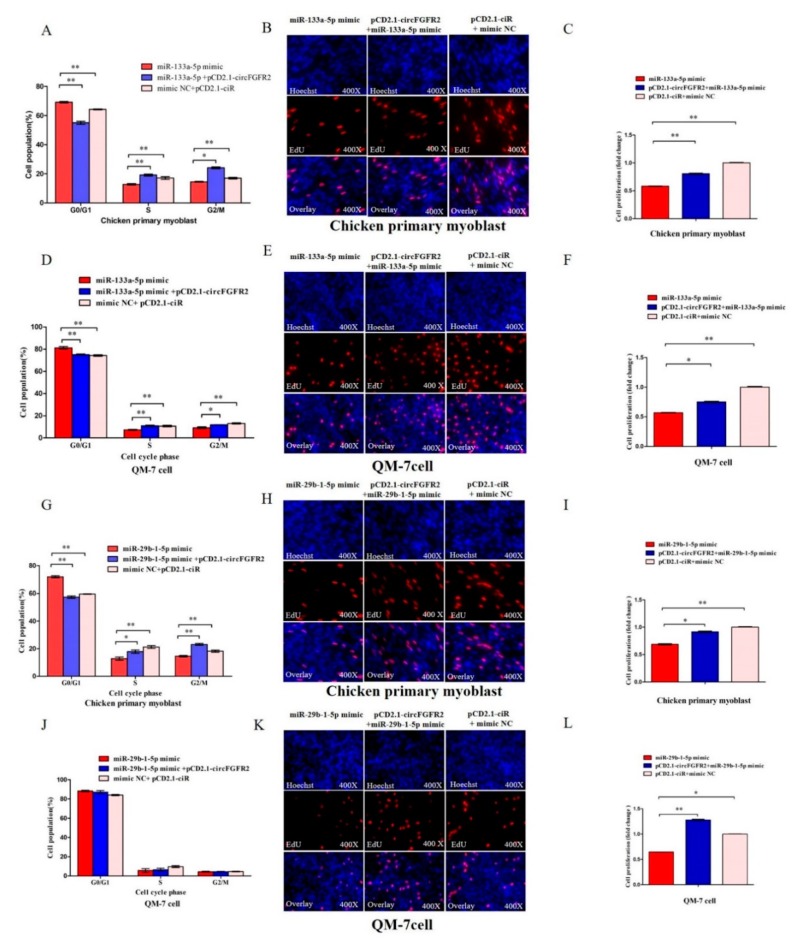
CircFGFR2 eliminates the inhibition effect of miR-133a-5p and miR-29b-1-5p on myoblast proliferation. (**A**) Cell cycle analysis of chicken primary myoblasts after co-transfection with the listed nucleic acids (miR-133a-5p, circFGFR2 overexpression vector and miR-133a-5p, empty overexpression vector and mimic NC, respectively) for 36 h. (**B**,**C**) EdU assays of chicken primary myoblasts after co-transfection with the listed nucleic acids (miR-133a-5p, circFGFR2 overexpression vector and miR-133a-5p, empty overexpression vector and mimic NC, respectively) for 36 h. (**D**) Cell cycle analysis of QM-7 cells after co-transfection with the listed nucleic acids (miR-133a-5p, circFGFR2 overexpression vector and miR-133a-5p, empty overexpression vector and mimic NC, respectively) for 48 h. (**E**,**F**) EdU assays of QM-7 cells after co-transfection with the listed nucleic acids (miR-133a-5p, circFGFR2 overexpression vector and miR-133a-5p, empty overexpression vector and mimic NC, respectively) for 48 h. (**G**) Cell cycle analysis of chicken primary myoblasts after co-transfection with the listed nucleic acids (miR-29b-1-5p, circFGFR2 overexpression vector and miR-29b-1-5p, empty overexpression vector and mimic NC, respectively) for 36 h. (**H**,**I**) EdU assays of chicken primary myoblasts after co-transfection with the listed nucleic acids (miR-29b-1-5p, circFGFR2 overexpression vector and miR-29b-1-5p, empty overexpression vector and mimic NC, respectively) for 36 h. (**J**) Cell cycle analysis of QM-7 cells after co-transfection with the listed nucleic acids (miR-29b-1-5p, circFGFR2 overexpression vector and miR-29b-1-5p, empty overexpression vector and mimic NC, respectively) for 48 h. (**K**,**L**) EdU assays of QM-7 cells after co-transfection with the listed nucleic acids (miR-29b-1-5p, circFGFR2 overexpression vector and miR-29b-1-5p, empty overexpression vector and mimic NC, respectively) for 48 h. In all panels, results are expressed as the mean ± S.E.M. of three independent experiments. For two group comparison analysis, statistical significance of differences between means was analyzed by unpaired Student’s *t*-test. For multiple comparison analysis, data were analyzed by one-way ANOVA followed by both least significant difference (LSD) and Duncan test through SPSS software. We considered *p* < 0.05 to be statistically significant. * *p* < 0.05; ** *p* < 0.01. NC, negative control.

Considering the interaction between circFGFR2 and miR-133a-5p/miR-29b-1-5p, rescue experiments were conducted by co-transfecting circFGFR2 with miR-133a-5p/miR-29b-1-5p mimics to assess whether the inhibition on proliferation of two miRNAs could be blocked by circFGFR2 overexpression. As expected, flow cytometry analysis and EdU assay confirmed that circFGFR2 could eliminate the inhibition from overexpressed miR-133a-5p (Figure 5A–F) or miR-29b-1-5p on the proliferation of both chicken primary myoblast and QM-7 cell (Figure 5G–L).

### 3.6. miR-133a-5p and miR-29b-1-5p Repress Myoblast Differentiation

**Figure 6:**
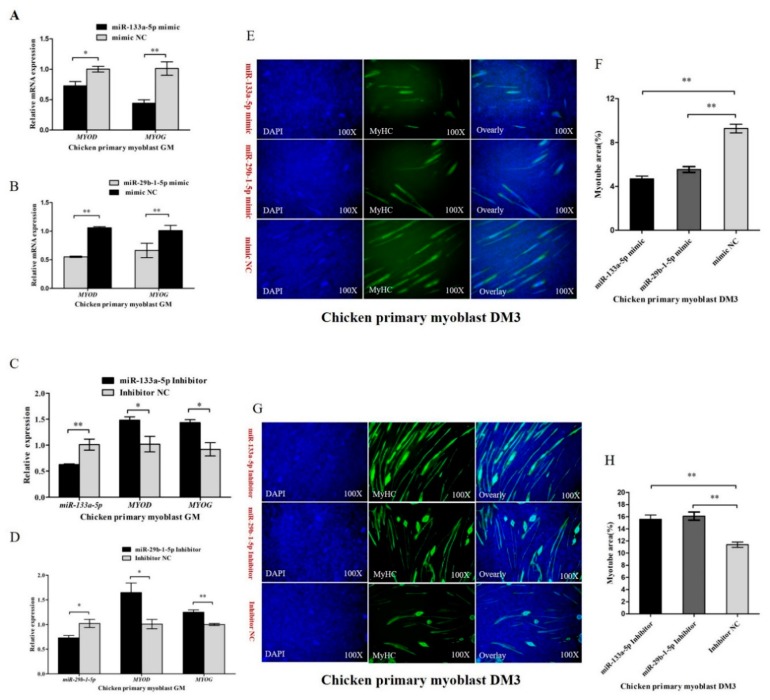
miR-133a-5p and miR-29b-1-5p repress myoblast differentiation. (**A**,**B**) Overexpression of miR-133a-5p and miR-29b-1-5p reduced the expression of *MYOD* and *MYOG*. (**C**,**D**) Inhibition of miR-133a-5p and miR-29b-1-5p accelerated the expression of *MYOD* and *MYOG*. (**E**,**F**) Immunofluorescence analysis of MyHC-staining cells after overexpression miR-133a-5p or miR-29b-1-5p. (**G**,**H**) Immunofluorescence analysis of MyHC-staining cells after down-regulation of miR-133a-5p or miR-29b-1-5p. In all panels, results are expressed as the mean ± S.E.M. of three independent experiments, and statistical significance of differences between means was assessed using an unpaired Student’s *t*-test (* *p* < 0.05; ** *p* < 0.01). NC, negative control.

To unveil the potential roles of miR-133a-5p and miR-29b-1-5p in chicken primary myoblast differentiation, the expression of the myoblast differentiation marker genes including *MYOG* and *MYOD* were evaluated by qRT-PCR in myoblast transfected with miR-133a-5p or miR-29b-1-5p. Overexpression of miR-133a-5p notably inhibited the expression of *MYOD* and *MYOG*, and overexpression of miR-29b-1-5p could also inhibit the expression of *MYOD* and *MYOG* (Figure 6A,B). Furthermore, we synthesized miR-133a-5p and miR-29b-1-5p inhibitor to down-regulate the expression of miR-133a-5p or miR-29b-1-5p, and we found that down-regulation of miR-133a-5p or miR-29b-1-5p accelerated the expression of *MYOD* and *MYOG* (Figure 6C,D). Subsequently, we induced chicken primary myoblast differentiation in vitro, and we transfected them with miR-133a-5p or miR-29b-1-5p mimic/inhibitor at DM1. MyHC immunofluorescence staining was carried out on the transfected differentiated myoblasts at DM3. According to immunofluorescence staining, we found that the total areas of myotubes of miR-133a-5p or miR-29b-1-5p mimic transfected group were prominently less than that of the control group (Figure 6E,F). On the contrary, the areas of myotubes in miR-133a-5p or miR-29b-1-5p (Figure 6G,H) inhibitor transfected group were more than that of the control group. The results demonstrated that miR-133a-5p and miR-29b-1-5p could repress chicken primary myoblast differentiation.

### 3.7. CircFGFR2 Eliminates the Inhibition Effect of miR-133a-5p and miR-29b-1-5p on Myoblast Differentiation

**Figure 7:**
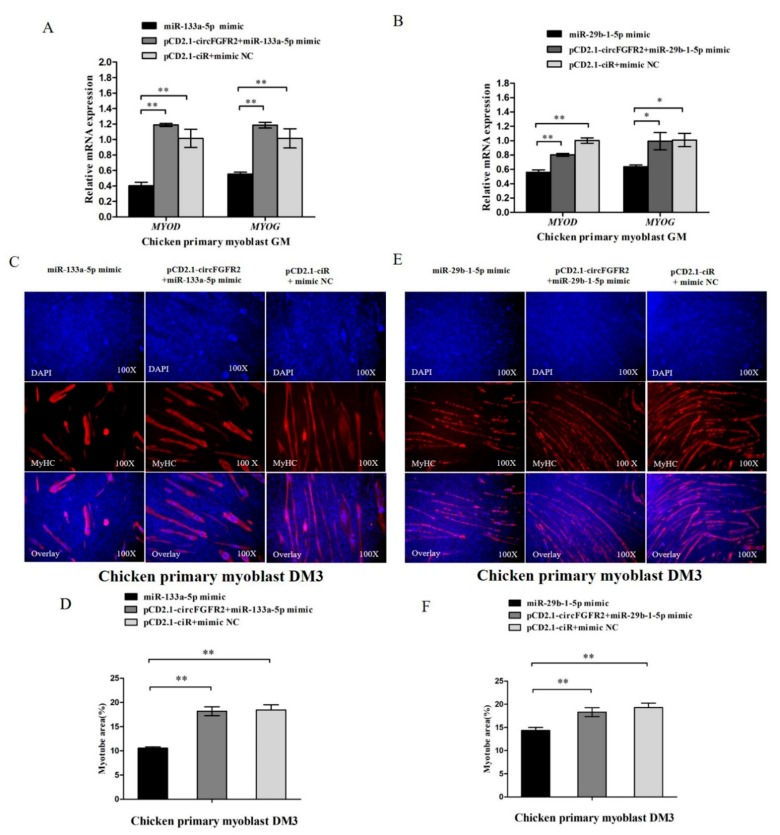
CircFGFR2 eliminates the inhibition effect of miR-133a-5p and miR-29b-1-5p on myoblast differentiation. (**A**) The mRNA expression of *MYOD* and *MYOG* of chicken primary myoblasts after co-transfection with the listed nucleic acids (miR-133a-5p, circFGFR2 overexpression vector and miR-133a-5p, empty overexpression vector and mimic NC, respectively). (**B**) The mRNA expression of *MYOD* and *MYOG* of chicken primary myoblasts after co-transfection with the listed nucleic acids (miR-29b-1-5p, circFGFR2 overexpression vector and miR-29b-1-5p, empty overexpression vector and mimic NC, respectively). (**C**,**D**) The myotube area of chicken primary myoblasts after co-transfection with the listed nucleic acids (miR-133a-5p, circFGFR2 overexpression vector and miR-133a-5p, empty overexpression vector and mimic NC, respectively). (**E**,**F**) The myotubes area of chicken primary myoblasts after co-transfection with the listed nucleic acids (miR-29b-1-5p, circFGFR2 overexpression vector, and miR-29b-1-5p, empty overexpression vector and mimic NC, respectively). In all panels, results are expressed as the mean ± S.E.M. of three independent experiments, and statistical significance of differences between means were analyzed by one-way ANOVA followed by both least significant difference (LSD) and Duncan test through SPSS software. We considered *p* < 0.05 to be statistically significant. * p < 0.05; * p < 0.01. NC, negative control.

We further performed a rescue experiment to investigate whether the suppressing effects of miR-133a-5p and miR-29b-1-5p on myoblast differentiation could be eliminated by circFGFR2 overexpression. As shown in Figure 7A, the expressions of *MYOD* and *MYOG* in miR-133a-5p and circFGFR2 co-transfected group were dramatically elevated compared with the miR-133a-5p transfected group. For miR-29b-1-5p, circFGFR2 also eliminated its repression effect on *MYOD* and *MYOG* (Figure 7B). Further MyHC immunofluorescence showed that overexpression of circFGFR2 eliminated the inhibition on myotube formation at DM3 caused by either miR-133a-5p or miR-29b-1-5p (Figure 7C–F). Taken together, these results demonstrated that circFGFR2 could eliminate the inhibition effect of miR-133a-5p and miR-29b-1-5p on myoblast differentiation.

## 4. Discussion

In recent years, circular RNAs have been successfully identified in various cell types across different species [7,9]. They have shown features of dynamic and tissue-specific expression, which indicate a distinct function in diverse tissues [45,46]. CircFGFR2 is a highly expressed DGcircRNA among millions of circRNAs during embryonic muscle development according to our previous circRNA sequencing results [22], which indicates that it has a potential effect in regulating skeletal muscle development. Here we primarily confirmed that circFGFR2 has a crucial function on skeletal muscle development. In both chicken primary myoblast and QM-7 cell, cell cycle analysis demonstrated that overexpression of circFGFR2 could significantly increase the cell numbers in S phase and reduce the cell numbers in G0/G1 phase, while downregulation of circFGFR2 showed the opposite effects. In addition, EdU incorporation assay confirmed that circFGFR2 elevated the cell proliferation rate as shown by overexpression and knockdown of circFGFR2. The results strongly supported that circFGFR2 could promote skeletal muscle cell proliferation. Skeletal myogenesis comes after cell cycle termination, which is coordinated by various regulatory transcription factors, including *MYOD*, *MYOG*, myogenic factor 5 (*Mrf5*), the muscle regulatory factor 4 (*Mrf4*), and myocyte enhancer factor-2 (*Mef2*) families [47,48]. *MYOD* and *MYOG* can regulate most myogenesis-related genes thus facilitating myoblast differentiation into myotubes [49,50]. *MyHC* is a differentiation marker gene of muscle and forms the backbone of the sarcomere thick filaments [51]. The circFGFR2 exerts a function in skeletal muscle cell proliferation, we detected whether circFGFR2 was also involved in skeletal muscle cell differentiation by monitoring the impact of circFGFR2 on the expression of *MYOD* and *MYOG*. As expected, circFGFR2 could promote the expression of *MYOD* and *MYOG*. MyHC immunofluorescence suggested that circFGFR2 accelerated the formation of myotubes, which confirmed another important role of circFGFR2 in skeletal muscle cell, i.e., it can facilitate myoblast differentiation.

Circular RNA is known to be a functional molecule transcribed from protein-encoding genes which contain MREs like other mRNAs or lncRNAs [52]. However, circular RNA was capable of escaping from degradation as it has no poly A tail could not be recognized by exonuclease compared with mRNAs or lncRNAs [5]. In addition, the expression level of some circular RNAs were not lower than their linear mRNAs [53]. Based on that advantage, they are efficient to act as ceRNA, which are enriched for stable miRNA binding sites and regulate the activity of miRNA. Bioinformatics technology is universally applicable for the analysis of the binding relationship of ceRNA and miRNA [54]. In this study, using the bioinformatics program RNAhybrid, we found that circFGFR2 had two possible binding sites for miR-133a-5p and one site for miR-29b-1-5p. Subsequently, we confirmed that miR-133a-5p and miR-29b-1-5p were actually combined with the predicated sites of circFGFR2 but not with *FGFR2* mRNA as indicated by two dual-luciferase reporter assays. Biotin-coupled miRNA pull down is an efficient method to verify the combined relationship between circular RNA and miRNA [18,19,55]. In this study, biotin-miR-133a-5p and biotin-miR-29b-1-5p were efficient in enriching circFGFR2, and overexpression of circFGFR2 significantly inhibits the expression of miR-133a-5p and miR-29b-1-5p which confirm the interacted relationship between circFGFR2 and miR-133a-5p/miR-29b-1-5p.

miR-133a-5p and miR-29b-1-5p belong to two miRNA families, miR-133 and miR-29, respectively. These two families have been well-studied miRNAs, and found to be involved in skeletal muscle cell proliferation and differentiation [27,38,56]. In mouse C2C12 cell line, miR-133 which contain a seed sequence of “UUGGUCC” could promote myoblast differentiation and inhibit cell proliferation, and miR-29 which contains a seed sequence of “AGCACCA” could reduce proliferation and facilitate differentiation [28,56]. The roles of miR-133a-5p and miR-29b-1-5p in avian skeletal muscle development still remain unclear. Here we first reported that miR-133a-5p and miR-29b-1-5p could repress the proliferation and differentiation of skeletal muscle cell. The roles of these two miRNAs were different from the studied miR-133 and miR-29 in mouse. We compared the sequence of miR-133a-5p and miR-29b-1-5p with other miR-133s and miR-29s in both chicken and mouse, and found that the mature sequences of gga-miR-133a-5p and gga-miR-29b-1-5p were different from the studied miR-133 and miR-29. Since the seed sequence was different, and miRNA exerts function by targeting the 3′-UTR of their target genes, it is possibly that the function of gga-miR-133a-5p and gga-miR-29b-1-5p was different from the miR-133 and miR-29 which have been studied in mouse. On the other hand, the roles of gga-miR-133a-5p or gga-miR-29b-1-5p were opposite to the effect of circFGFR2 in myoblast. It is therefore reasonable that circFGFR2 could act as a molecular sponge for miR-133a-5p and miR-29b-1-5p. To confirm this, we further performed rescue experiments and found that circFGFR2 eliminated the inhibition effect of miR-133a-5p and miR-29b-1-5p on myoblast proliferation and differentiation. Considering all of this, we declared that circFGFR2 regulates skeletal muscle cell proliferation and differentiation by inhibiting the expression and activity of miR-133a-5p and miR-29b-1-5p in poultry.

## 5. Conclusions

In conclusion, we found that a novel circular RNA of circFGFR2, generated by the FGFR2 gene, could regulate myoblast proliferation and differentiation by acting as a sponge of miR-133a-5p and miR-29b-1-5p in poultry.

## Figures and Tables

**Table 1:** Primers used in this study.

Name	Nucleotide Sequences (5′→3′)	Annealing Temperature (°C)	Size	Application
circFGFR2	F: ACATCGTATTGGCGGCTAT	60	267	qRT-PCR for circFGFR2
	R: ACCCCATCCTTAGTCCAAC			
FGFR2-1	F: GTCCGCTGTATGTGATTGTAG	56	129	qRT-PCR for FGFR2 gene
	R: TGAATGTCATCTGCTCCTCT			
FGFR2-2	F: AGCCGCCAACCAAATACCAAATR: CGACAACATCGAGATGGTAAGT	56	636	Amplification of the whole linear sequence of circFGFR2
MYOD	F: GCTACTACACGGAATCACCAAAT	58	200	qRT-PCR
	R: CTGGGCTCCACTGTCACTCA			
MYOG	F: CGGAGGCTGAAGAAGGTGAA	60	320	qRT-PCR
	R: CGGTCCTCTGCCTGGTCAT			
β-actin	F: ACCACAGGACTCCATACCCAAGAAAG	52–60	146	qRT-PCR
	R: GCCGAGAGAGAAATTGTGCGTGAC			
